# Postoperative full abduction in a patient of Duane retraction syndrome without an abducens nerve: a case report

**DOI:** 10.1186/s12886-017-0475-6

**Published:** 2017-05-19

**Authors:** Jae Hyoung Kim, Jeong-Min Hwang

**Affiliations:** 1 Department of Radiology, Seoul National University College of Medicine, Seoul National University Bundang Hospital, Seongnam, Korea; 2 Department of Ophthalmology, Seoul National University College of Medicine, Seoul National University Bundang Hospital, 166, Gumiro, Bundang-gu, Seongnam, Gyeonggi-do 463-707 Korea

**Keywords:** Duane retraction syndrome, Postoperative full abduction, Abducens nerve

## Abstract

**Background:**

Duane retraction syndrome (DRS) consists of abduction deficit, globe retraction and upshoots or downshoots with adduction. The abducens nerve on the affected side is absent in type 1 DRS. After bilateral medial rectus muscle recession in unilateral type 1 DRS may improve the abduction limitation, but still more than −3 limitation remains.

**Case presentation:**

A 6-month-old boy presented with esotropia which had been noticed in early infancy. He showed limited abduction, fissure narrowing on attempted adduction and a small upshoot OS. Left abducens nerve was not identified on magnetic resonance imaging compatible with Duane retraction syndrome type 1. He showed full abduction after bilateral medial rectus recession of 6.0 mm at the age of 9 months, and remained orthotropia with full abduction OU 2 years postoperatively. He is my only patient with Duane retraction syndrome who showed full abduction after bilateral medial rectus recession.

**Conclusions:**

A patient with the type 1 Duane retraction syndrome rarely may show full abduction after bilateral medial rectus recession mimicking infantile esotropia.

## Background

Duane retraction syndrome (DRS) consists of abduction deficit, globe retraction and upshoots or downshoots with adduction [1]. The abducens nerve on the affected side is absent in type 1 DRS and part of type 3 DRS [2]. Vertical rectus muscle transposition with/without augmented suture, superior rectus transposition with/without augmented suture or medial rectus recession, or uni−/bilateral medial rectus muscle recession alone in uni−/bilateral type 1 DRS have been reported to improve the abduction limitation [3–13], but still more than −1 limitation remains at the best after vertical rectus muscle transposition with or without augmented suture [13]. We observed a patient who showed full abduction after bilateral medial rectus muscle recession in spite of the absent abducens nerve in the left.

## Case presentation

A 6-month-old boy presented with esotropia which had been noticed in early infancy. He was born at the age of 38 weeks with a birth weight of 3.2 kg. The delivery and perinatal period was uneventful. He did not have a family history of strabismus.

**Fig. 1:**
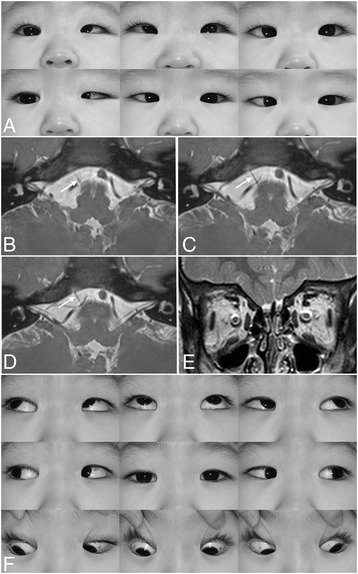
Duane retraction syndrome type 1 in the left. **a** Ocular versions demonstrating limited abduction, fissure narrowing on attempted adduction and a small upshoot in the left eye. **b**-**d** Three consecutive thin-section T2-weighted images show the normal right abducens nerve (arrows) as a dark structure emerging from the pontomedullary junction (**b**), coursing superiorly and obliquely (**c**), and finally entering the Dorello’s canal (**d**). The left abducens nerve is not identified. **e** Coronal T2-weighted image of the orbit shows extraocular muscles in normal size. **f** Ocular versions demonstrating full abduction and a small upshoot in the left eye

On ophthalmological examination, he fixed and followed a 5-inch toy with either eye, better with the left eye. Krimsky method showed 35 prism diopters (PD) of right esotropia in the primary position (Fig. 1a). He showed limited abduction (Fig. 1a), palpebral fissure narrowing on attempted adduction and a small upshoot in the left eye. Cycloplegic refraction showed +3.50 diopters (D) sph in the right eye and +3.25 Dsph in the left eye. No additional abnormal findings were discovered following slit lamp and fundus examination. Glasses to fully correct the hyperopia, 7.5 PD base out prisms in both eyes and occlusion of the right eye 30 min a day were prescribed.

MRI was conducted using a 3 tesla system (Intera achieva; Philips Medical Systems, Best, the Netherlands) with a SENSE head coil. Thin-section T2-weighted imaging was performed at the brainstem with 1.4 mm of section thickness for the abducens nerve and the oculomotor nerve, and with 0.25 mm of section thickness for the trochlear nerve. Coronal T2-weighted imaging of the orbit was performed with 2 mm of section thickness. Left abducens nerve was not identified and extraocular muscles in both orbits were normal in size (Fig. 1b-e).

One month later, esotropia decreased to 10 PD with correction in the primary position with alternate prism cover test. Two months later, esotropia increased to 30 PD, and further increased to 40 PD 3 months later. He underwent bilateral medial rectus recession of 6.0 mm at the age of 9 months. He remained orthotropia with full abduction in both eyes (Fig. 1f) 2 years postoperatively.

## Discussion

Differential diagnoses in children with abduction limitation include DRS, abducens nerve palsy, Ciancia syndrome [14] and infantile esotropia. MRI could be helpful because abducens nerve is absent in type 1 DRS, and present in abducens nerve palsy or infantile esotropia [2, 15]. Atrophy of the lateral rectus muscle may be the only finding in abducens nerve palsy [15]. We considered the above mentioned differential diagnosis in our patient. However, we consider at least reasons to believe our patient had DRS.

Firstly, postoperative abduction was unexpectedly too full, thus we checked the presence of the abducens nerve in MR imaging again and again. There was definitely no abducens nerve. Therefore, the diagnosis of infantile esotropia or Ciancia syndrome would be inappropriate. Secondly, Ciancia syndrome is characterized by large angle esotropia, jerk nystagmus in abduction, latent nystagmus, torticollis and asymmetric optokinetic nystagmus [14]. Our patient showed neither jerk nystagmus in abduction nor torticollis, and the esotropia was not that large (35 PD). Thirdly, before the surgery, either eye was patched and abduction was looked for in either eye. Parents were also asked to patch either eye and take photographs of the non-patched eye. In the clinic as well as at home, the right eye showed full abduction, but the left eye did not, which is not usual for infantile esotropia or Ciancia syndrome. Finally, our patient showed limited abduction, fissure narrowing on attempted adduction and a small upshoot in the left eye.

Diagnosis of DRS in children could be difficult because the diagnostic signs of DRS, such as severe retraction on adduction, upshoot, and downshoot, may not be manifest [16–18]. The incidence of palpebral fissure narrowing on adduction, upshoots and downshoots is significantly lower in children than in adults with DRS, because the lateral rectus muscle becomes more fibrotic with aging [16–18]. Caputo et al. [17] reported that correct diagnosis diagnosis of DRS in children with DRS was 54% by age 14 months and to increase to 87.5% at 35 months. Therefore, children with only abduction deficit can be challenging, and MR imaging could be more helpful for the differential diagnosis of abduction deficit in children. This patient showed fissure narrowing on attempted adduction and a small upshoot in the left eye which were not marked, and could be misdiagnosed with inferior oblique overaction frequently accompanied by infantile esotropia.

The degree of post-operative improvement following the medial rectus recession in a patient with DRS might be variable depending on the tightness of medial rectus muscle. Farvardin et al. [3] reported improved abduction limitation after bilateral medial rectus muscle recession in unilateral type 1 DRS, but still had −3 limitation. Foster [4] reported −3 to −3.5 abduction limitation after full vertical rectus muscle transposition with augmented (Foster) suture in 5 patients with DRS. Velez et al. [5] reported residual abduction limitation of −2.9 ± 0.6 after transposition of both vertical rectus muscles with a posterior augmentation suture and −2.7 ± 0.7 after same procedure without a posterior augmentation suture. Rosenbaum [6] reported improvement in abduction from −3.9 to −2.8 after augmented transposition of the vertical rectus muscles in 42 patients with unilateral DRS, and-3.7 to −2.6 after non-augmented transposition of the vertical rectus muscles in 22 patients with unilateral DRS. Yazdian et al. [7] improved abduction limitation from −4.00 ± 0.23 to −2.11 ± 0.48 after augmented transposition of the vertical rectus muscles in 38 patients with type 1 DRS. Britt et al. [8] reported −2.5 to −4.0 abduction limitation after partial vertical rectus muscle transposition with augmented suture in 5 patients with DRS. Sterk et al. [9] reported improvement of abduction after both vertical muscle transposition and medial rectus recession in patients with DRS type I by 15.9° ± 8.1° on average. More recently, Mehendale et al. [10] reported improved abduction from −4.3 to −2.7 after superior rectus transposition combined with medial rectus recession in 10 patients with DRS and 7 with sixth nerve palsy. Yang et al. [11] found that a combination of superior rectus transposition and medial rectus recession was more effective than medial rectus recession alone for improving abduction. The average improvement of abduction was −4 to −2.5 with superior rectus transposition, and −3.5 to −3 without superior rectus transposition by the Figure. Tibrewal et al. [12] also compared the results of augmented superior rectus transposition with or without medial rectus recession. The average improvement of abduction was from −3.6 to −2.4 with superior rectus transposition, and −3.6 to −3.3 with medial rectus recession. The best result was reported by Britt et al. [13], of −1 after augmented transposition of the vertical rectus muscles in 1 patient with bilateral DRS. Therefore, the remaining abduction limitation may be −1 at the best after vertical rectus muscle transposition with or without augmented suture.

We had two more patients who showed full abduction in spite of the bilaterally absent abducens nerve [19, 20]. In addition, both of them did not show trochlear nerves bilaterally [19, 20]. However, this patient showed full abduction only after bilateral medial rectus recession, and trochlear nerves were identified. Type 2 DRS patient could show full abduction, but they have the abducens nerve [2, 21]. This patient showed full adduction, and he did not show the abducens nerve, therefore, not compatible with type 2 DRS. To the best of our knowledge, this is the first report of a patient with type 1 DRS who showed full abduction after bilateral medial rectus recession. We had one more patient who showed full abduction only with maximal effort [22]. He mostly showed both limitation of adduction and abduction and did not show abducens nerve in brain MRI. However, this patient did not show significant abduction with repeated examination both in the clinic and at home with patching of the contralateral eye. Therefore, this patients is not similar to the patient in the previous report [22].

It is difficult to explain this dramatic improvement in abduction in this patient. Possible mechanisms could be increased synergistic innervations to left lateral rectus through the aberrant innervations to lateral rectus after weakening of the right medial rectus as a part of bilateral medial rectus recession. There is the possibility of development of fixation duress due to contralateral medial rectus recession that has led to decreased innervational drive to ipsilateral medial rectus. In postoperative period, reduced innervations to ipsilateral medial rectus would reduce the likelihood of repeat contracture of the medial rectus that would contribute to improved abduction. Another could be an acquired new aberrant innervation to lateral rectus muscle. Lastly, preexisting aberrant innervation ot the lateral rectus muscle could work after releasing the restriction by the tight medial rectus. More studies looking at the imaging of abducens nerve and its nucleus might shed more light in such cases.

## Conclusion

A patient with the type 1 DRS rarely might show full abduction after bilateral medial rectus recession mimicking infantile esotropia. Future studies looking at the imaging of abducens nerve and its nucleus might shed more light in such cases.

## Abbreviations

D: Diopters
DRS: Duane retraction syndrome
PD: Prism diopters
